# Motor Imagery Classification Using Effective Channel Selection of Multichannel EEG

**DOI:** 10.3390/brainsci14050462

**Published:** 2024-05-03

**Authors:** Abdullah Al Shiam, Kazi Mahmudul Hassan, Md. Rabiul Islam, Ahmed M. M. Almassri, Hiroaki Wagatsuma, Md. Khademul Islam Molla

**Affiliations:** 1 Department of Computer Science and Engineering, Sheikh Hasina University, Netrokona 2400, Bangladesh; shiam.cse@shu.edu.bd; 2 Department of Computer Science and Engineering, Jatiya Kabi Kazi Nazrul Islam University, Trishal, Mymensingh 2224, Bangladesh; kmhassan@jkkniu.edu.bd; 3 Department of Medicine, University of Texas Health Science Center, San Antonio, TX 78229, USA; rabiul@sip.tuat.ac.jp; 4 Department of Intelligent Robotics, Faculty of Engineering, Toyama Prefectural University, Toyama 939-0398, Japan; almassri@pu-toyama.ac.jp; 5 Department of Human Intelligence Systems, Graduate School of Life Science and Systems Engineering, Kyushu Institute of Technology, Fukuoka 808-0196, Japan; waga@brain.kyutech.ac.jp; 6 Department of Computer Science and Engineering, The University of Rajshahi, Rajshahi 6205, Bangladesh

**Keywords:** brain–computer interface, channel selection, electroencephalography, entropy-based information, motor imagery

## Abstract

Electroencephalography (EEG) is effectively employed to describe cognitive patterns corresponding to different tasks of motor functions for brain–computer interface (BCI) implementation. Explicit information processing is necessary to reduce the computational complexity of practical BCI systems. This paper presents an entropy-based approach to select effective EEG channels for motor imagery (MI) classification in brain–computer interface (BCI) systems. The method identifies channels with higher entropy scores, which is an indication of greater information content. It discards redundant or noisy channels leading to reduced computational complexity and improved classification accuracy. High entropy means a more disordered pattern, whereas low entropy means a less disordered pattern with less information. The entropy of each channel for individual trials is calculated. The weight of each channel is represented by the mean entropy of the channel over all the trials. A set of channels with higher mean entropy are selected as effective channels for MI classification. A limited number of sub-band signals are created by decomposing the selected channels. To extract the spatial features, the common spatial pattern (CSP) is applied to each sub-band space of EEG signals. The CSP-based features are used to classify the right-hand and right-foot MI tasks using a support vector machine (SVM). The effectiveness of the proposed approach is validated using two publicly available EEG datasets, known as BCI competition III–IV(A) and BCI competition IV–I. The experimental results demonstrate that the proposed approach surpasses cutting-edge techniques.

## 1. Introduction

A brain–computer interface (BCI) is a system that enables humans to communicate with the environment without depending on a peripheral limb or muscle [1,2]. BCI can assist people in carrying out regular tasks without moving their bodies. BCI applications extend from communication and rehabilitation to entertainment [3]. Noninvasive methods such as functional magnetic resonance imaging (fMRI), magnetoencephalography (MEG), and electroencephalography (EEG) can be used to monitor brain activity. The EEG-based BCI application has become the most popular because it is very easy to implement and is cost-effective [4,5,6,7,8]. A common form of mental task employing EEG-based BCI is motor imagery (MI), which demands users to articulate particular limb or muscle movements without performing actual action. Event-related synchronization (ERS) and event-related desynchronization (ERD), which MI-BCI systems may detect, are oscillatory activity in sensorimotor rhythms that are produced by these imaginations [9,10,11]. The MI classification using multichannel EEG is investigated in this study to identify MI-related brain activity patterns. The aim of this work is to improve the performance of BCI systems. Although there is a noticeable advancement in the study of MI classification using EEG, its performance is affected by the high dimensionality of EEG, intersubject variability, and noise. Moreover, it is still not clear how well the conventional design of channels captures brain signals that are effective for MI categorization [12]. This study focuses on the selection of optimal channels for MI classification using multichannel EEG to overcome the traditional limitation. The channels with redundant and noisy information are discarded such that the MI classification is improved. Rigorous experiments are conducted to demonstrate the efficacy of the proposed method.

An entropy-based channel selection mechanism is introduced to implement the MI classification system in this paper. The EEG signals with a reduced number of channels are decomposed into a finite number of sub-bands. The common spatial pattern-based features are calculated from the sub-bands and used for the classification.

The rest of this paper is organized as follows: Section 2 discusses the related works that already have been conducted on MI classification; Section 3 describes the data used in this study; Section 4 explains the technique; Section 5 illustrates the experimental results; and Section 6 and Section 7 include the discussion and some concluding remarks, respectively.

## 2. Related Works

For a variety of applications, many researchers have been striving to create EEG-based BCI systems [12,13,14]. For better performance, most BCI systems gather EEG inputs from several scalp electrodes. However, it takes a lot of time and effort to set up a BCI system with the sensors. Additionally, it is a significant obstacle to integrating BCI into commonplace applications [13,15,16]. A survey includes new frontiers in applying deep learning for brain signal analysis [17]. Furthermore, the presence of large EEG channels introduces issues such as distortion, excessive computational complexity, and data redundancy [18,19]. Therefore, while designing BCI systems, the selection of a reduced number of effective channels becomes demanding. Several approaches have been introduced to select the effective number of EEG channels. Some techniques rank the channels under independent evaluation standards without employing any classifier. In contrast, other methods evaluate the significance of channels by considering the effectiveness of particular classifiers [20].

Graph Sequence Neural Network (GSNN) for accurately decoding patterns of motor imagery from EEGs is proposed in [21]. The model utilizes a node domain attention selection network. It focuses on training sample datasets specific to each subject at different distraction themes, adapting to subjects’ influences, and reducing noise samples [21]. Specifically, it illustrates the difficulty in deciding between a global pooled architecture and a hierarchical pooled architecture for graph classification in MI (Motor Imagery). The errors of linear discriminant analysis (LDA) classifier test have been employed in [22] as criteria to select potential EEG channels. These approaches have not achieved the required level of classification accuracy. A correlation-based channel selection with a regularized common spatial pattern (CCS-RCSP) is implemented in [23] for MI classification. In order to correctly identify the MI tasks, a support vector machine (SVM) classifier with the radial basis function (RBF) kernel is used. In [24], a set of the relevant channels are selected using the binary gravitational search algorithm (BGSA). A bandpass filter was used there to reduce the noise. Additionally, artifacts like electromyography (EMG) and electrooculography (EOG) are eliminated using the blind source separation (BSS) technique. Signals are then processed in time and wavelet domains to extract features. The efficiency of their channel selection strategy is then evaluated using the SVM classifier.

In [25], a unique channel selection method based on the correlation coefficient (CSCC) is used. For each channel, a collection of uncorrelated channels is chosen. To pick one of these groups, the Fisher score is computed after extracting the features of each group using filter-bank CSP (FBCSP). A group of channels is then chosen which has the highest Fisher score. A channel selection approach is suggested in [19] using the spatiotemporal information in EEG data. The selection process has been conceptualized as an optimization problem. It is solved via a computationally efficient approach by incorporating sparsity restrictions.

The common spatial pattern (CSP)-based method is a known technique for EEG channel selection but it is greatly affected by the frequency spectrum and temporal window of the EEG segments. In [26], the time-constrained sparse group spatial pattern (TSGSP) method is introduced to improve CSP. The effects of frequency and temporal events are taken into account concurrently. A cross-correlation-based discriminant criterion (XCDC) is proposed in [27] to measure the significance of a channel for differentiating the mental states of various motor imagery (MI) tasks. The chi-square statistics is used to select a subset of channels in [28]. The sparse common spatial pattern (SCSP) approach is used to choose the MI-relevant channels that correlate to high sparse CSP filter coefficients [18]. Using the least absolute shrinkage and selection operator (LASSO) algorithm, the CSP-rank for multiple frequency bands (CSP-R-MF)-based approaches suggest in [29] to pick the MI-relevant channels for each frequency band.

The effective EEG channels (DCRCC) are identified in [30] using a dynamic channel relevance (DCR) score. The lowest redundancy maximum relevance paradigm is introduced for choosing the appropriate channels. The Savitzky–Golay filter is used to preprocess the EEG signals, and the sliding window method is employed to divide the resulting segments into predetermined lengths. Three previously identified channels are also considered as possible options for discovering additional related channels. The variance ratio dispersion score (VRDS) and interclass feature distance (ICFD) of small EEG channel groups are two metrics used in the local region CSP (LRFCSP) method proposed in [31] to choose channels. The robust feature selection (RFS) algorithm used in the feature compression and channel ranking (FCCR) approach selects the MI-relevant channels by clustering the feature dimension [32]. These EEG channel selection methods rely on temporal, spectral, or spatial characteristics. It is used to classify MI tasks in relation to regionally different ERD/ERS patterns. However, when categorizing delicate and mixed ERD/ERS patterns, these traits may have limitations.

The existing methods for channel selection exhibit relatively poor classification performance because they often fail to accurately identify and eliminate all deceptive channels. In several studies, the number of optimized features is still large, which is less informative and error-prone. Techniques based on DCR score lack candidate channels, which causes performance degradation. In this paper, an entropy score used for effective EEG channel selection is proposed to improve MI classification performance. It mitigates the mentioned limitations of the existing methods. The entropy quantifies the pattern’s unpredictability or uncertainty, which is roughly comparable to the signal’s information content. High entropy means a more disordered pattern, whereas low entropy means a less disordered pattern with less information. The Shannon entropy is computed for each of the channels to obtain entropy scores. Then, a group of high-entropy channels for further processing are dynamically selected. Later, filtering techniques are applied to selected channels to decompose into different frequency bands. Using CSP, the spatial characteristics are extracted from each sub-band and fused to create the feature vector. The EEG features derived from the effective channels are fed to the support vector machine (SVM) classifier. The effectiveness of the suggested approach is assessed using an EEG dataset that is freely accessible. To demonstrate the superiority of the EEG channel selection methodology used in this study, the experimental findings are contrasted with the MI classification methods that are currently in use.

## 3. Data Description

### 3.1. Dataset 1

A publicly available dataset, BCI Competition III (IVA), is used in this study to evaluate the proposed approach. The dataset was received from the Berlin BCI group. It includes the Campus Benjamin Franklin of the Charité, Department of Neurology, Neurophysics Group (Gabriel Curio), and Fraunhofer FIRST, Intelligent Data Analysis Group (Klaus-Robert Müller, Benjamin Blankertz). A detailed description of the dataset is available in [33]. Five healthy subjects (aa, al, aw, av, and ay) aged 24 to 25 completed MI tasks on each trial to record the dataset as EEG signals. They were instructed not to move their hands or arms during the recordings and not to move their eyes. The dataset consists of four sessions, each with 70 runs, for a total of 280 runs per subject. Out of 280 runs, 168, 224, 84, 56, and 28 runs for subjects aa, al, aw, av, and ay, respectively, made up the training set, whereas the remaining runs made up the test set. During the 3.5 s that the visual cues suggested, respondents were instructed to carry out one of two motor imagery tasks: right hand or right foot movement. One of the aforementioned activities was given to the users to consider, with goal cue intervals of 1.75 to 2.25 s, during which the subject might unwind. The 128-channel Ag/AgCl electrode cap was used to record the EEG signals. Positions were measured for 118 EEG channels using the worldwide 10/20 system. The bandpass filter was used to filter the measured MI-based EEG signals between 0.05 and 200 Hz, and the signals were subsequently digitized at 1000 Hz with a 16-bit precision. The dataset is downsampled at 100 Hz for further processing. Table 1 represents the summary of Dataset 1.

### 3.2. Dataset 2

**Figure 1:**
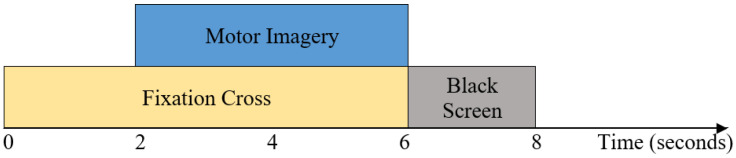
The timing sequence of BCI competition IV dataset I (Dataset 2).

The second dataset used in this study is obtained from BCI Competition IV Dataset I [34], which is also publicly available. The summary of Dataset 2 is presented in Table 2. There are seven healthy subjects, namely a, b, c, d, e, f, and g. The EEG signals were recorded using 59 channels. The sampling frequency of Dataset 2 is 100 Hz. The left hand, right hand, and foot are the three classes of motor imagery tasks that are involved. Each subject completed any two of the classes. Four seconds of visual cues in the shape of arrows were displayed on the screen, and during that time, the subject completed a specific mental task. The signals were interspersed with two seconds of a blank screen and two seconds of a fixation cross. The fixation cross was superimposed on the cues and shown for six seconds. Thus, each trial lasts for 4 s, and then there is a 4 s rest interval. There are 200 trials in total for each subject and 100 trials for each of the two classes that are chosen by the particular subject. A trial lasts 4 s, and there are 400 samples in a trial (4 s × 100 Hz). Different parameters of Dataset 2 are illustrated in Figure 1.

## 4. Methodology

**Figure 2:**
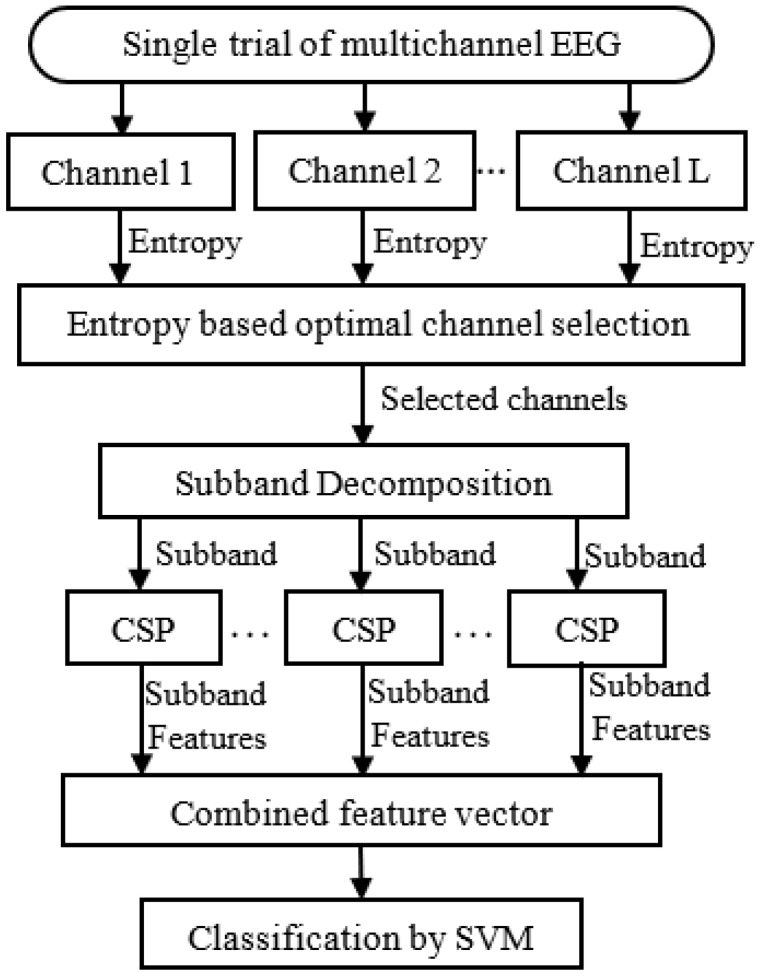
Block diagram of the proposed MI classification system based on channel selection.

The number of channels is increased day by day to enhance the spatial resolution of EEG-based BCI implementation. All the channels used in the recording of EEG are not effectively usable in different applications. The issue for selecting application-dependent effective channels is the demand to improve EEG classification performance. Such a method is implemented in this study. The methodology starts with the calculation of Shannon entropy for each channel in a trial. The entropy measure is used to identify channels having significant information. Based on the entropy scores, an optimal number of effective channels are selected for further analysis. Subsequently, the EEG signals with the selected channels are decomposed into distinct sub-bands. The narrowband approach enables the features of frequency-specific components of the EEG data. The sub-band trials are generated by aggregating similar sub-bands obtained from all selected channels. Common spatial pattern (CSP)-based features are then extracted from the sub-band trials. A feature vector is constructed by combining the features obtained from different sub-bands. The vector encapsulates the relevant information extracted from the EEG data. Finally, classification is performed using a support vector machine (SVM). The block diagram of the proposed channel-selection-based approach is illustrated in Figure 2. The following steps summarize the proposed method:

- The entropy of each channel of a trial is calculated. The Shannon entropy is used here.
- The obtained entropy score is used to select the adequate number of channels.
- The EEG signals of the selected channels are decomposed into a finite set of sub-bands using the Butterworth bandpass filter.
- Sub-band trial is generated by an accumulated similar sub-band obtained from all of the selected channels. Common spatial pattern (CSP)-based features are extracted from the sub-band trial.
- A feature vector is formulated by combining the features extracted from each sub-band trial.
- The classification is performed by implementing a support vector machine (SVM) along with the feature vector.

### 4.1. Data Preprocessing

**Figure 3:**
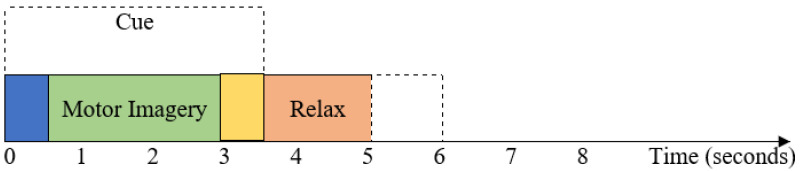
The timing sequence of the BCI competition III dataset IVA (Dataset 1).

For Dataset 1, the visual cues are illustrated for 3.5 s, as shown in Figure 3. The EEG signals from 0.5 s to 3 s after the cue are used in this study. Pre- and postimagination are thought to last for the first 0.5 s (0–0.5 s) and the last 0.5 s (3–3.5 s), respectively. For Dataset 2, the visual cues are illustrated for 4 s, as shown in Figure 1. The EEG signals from 2 s to 6 s after the cue are used in this study for Dataset 2. Then, the EEG signal of both datasets is passed through a fifth-order Butterworth bandpass filter with a frequency range from 8 Hz to 35 Hz.

### 4.2. Effective Channel Selection

All the EEG channels are not equally effective for motor imagery (MI) classification. Efficient channel selection considerably improves the classification accuracy of MI tasks. An entropy-based information-theoretic approach is implemented here to select the effective EEG channels. The multichannel EEG is denoted as $X_{l}^{m}={\left[x_{l}^{m}\left(1\right)x_{l}^{m}\left(2\right)\ldots x_{l}^{m}\left(N\right)\right]}^{T}$, where *m* is the trial index (m=1,2,…,M), *l* is the channel index (l=1,2,…,L), and *n* is the index of sample (n=1,2,…,N). The entropy measures the information of the pattern [35,36,37,38,39]. The Shannon entropy score is used in this study to identify the effective EEG channels. For a specific single subject, the average entropy of the *l*-th channel over *M* trials is defined as

(1) $$h_{l}=-\frac{1}{M}\sum_{m=1}^{M}\sum_{n=1}^{N}p\left(x_{l}^{m}\left(n\right)\right)\log p\left(x_{l}^{m}\left(n\right)\right),$$

where $p(x_{l}^{m}\left(n\right))$ is the probability of the occurrence of the *n*-th sample on the *l*-th channel of the *m*-th trial. The entropy vector $H=[h_{1},h_{2},\ldots .,h_{l},\ldots ,h_{L}]$ is sorted in descending order, and the channels with higher entropy scores are chosen as the effective channel to improve MI classification performance. The EEG data of reduced channels consisting of the selected channels only are denoted by $X^{\prime }$.

### 4.3. Sub-Band Decomposition

**Figure 4:**
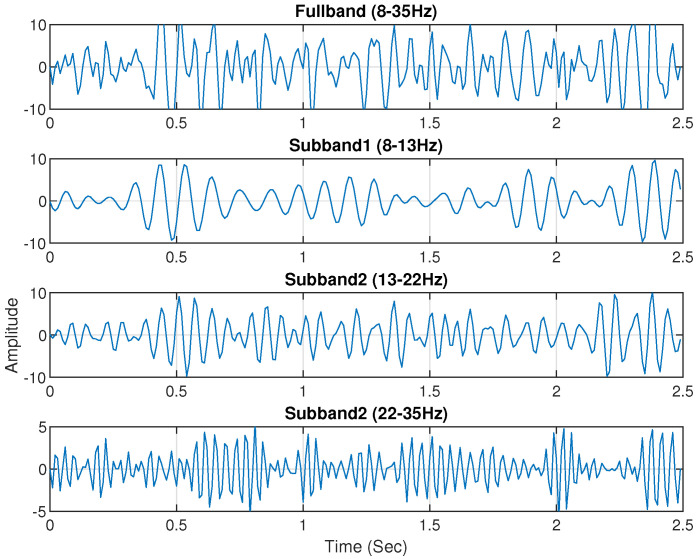
Channel C3 of Subject ‘al’ from the BCI Competition III IVA dataset yielded four unique sub-bands: 8–35 Hz, 8–13 Hz, 13–22 Hz, and 22–35 Hz.

**Figure 5:**
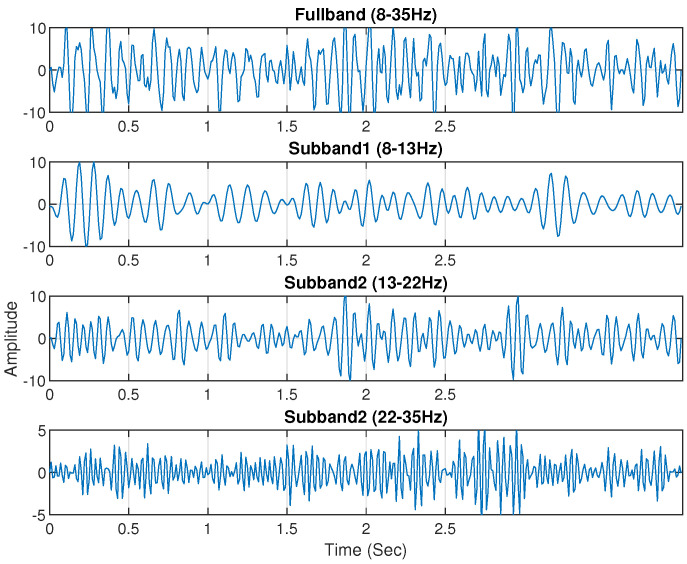
The four sub-bands: 8–35 Hz, 8–13 Hz, 13–22 Hz, and 22–35 Hz of channel C3 selected from Subject ‘a’ of BCI Competition IV dataset I.

Electrophysiological noises frequently contaminate the multichannel raw EEG. Furthermore, some narrowband components of the EEG signal respond more strongly to the specific MI task. Therefore, the use of sub-band signals of selected EEG channels would be more accurate in classifying MI activities. According to a previous study, the majority of brain activities connected to MI tasks take place at the frequency range of 7–30 Hz [40,41]. Then, the EEG signal is split into four sub-bands using a zero-phase Butterworth bandpass filter. The extracted four sub-bands are Mu (8–13 Hz), low beta (13–22 Hz), high beta (22–35 Hz), and full-band (8–35 Hz). Figure 4 and Figure 5 shows the sub-bands of channel C3 for subject ‘al’ and ‘a’ of the BCI Competition III (IVA) dataset and BCI Competition IV (I) dataset.

### 4.4. Feature Extraction

The potential feature extraction is one of the most important steps in BCI implementation. The features have a direct influence on the BCI system performance. Recent studies have looked at modifying or developing feature extraction algorithms [42]. The most popular and well-known feature extraction technique in multichannel EEG-based BCI systems is the CSP [41,43,44]. Multichannel EEG data are projected onto a low-dimensional spatial subspace using the CSP technique. The CSP determines spatial filters that maximize the variance ratio between two classes of data. The filter serves to extract effective features for classification. The most discriminative spatial information between the MI tasks to execute the effective classification of EEG signals is applicable in BCI implementation. Let $X_{c_{1}}^{\prime }$ and $X_{c_{2}}^{\prime }$ denote the training trials of EEG signals with selected effective channels from class c1 and c2, respectively. The CSP method derives the features by simultaneously diagonalizing both classes’ covariance matrices. The EEG data are transformed into a projection matrix using a spatial filter $w\in R^{l}$ that maximizes the variances between two classes [45].

(2) $$w=\arg\max\frac{w^{T}B_{1}w}{w^{T}B_{2}w}\;s.t\;{∥w∥}_{2}=1.$$

By resolving an extended eigenvalue problem, the optimal solution to Equation (2) is achieved. The largest and lowest eigenvalues *V* generated by the eigenvectors are included in the spatial filter $w=\left[w_{1},w_{2},\ldots ,w_{2V}\right]\in R^{l\times 2V}$. Thus, the feature vector of any trial of sub-band *s* is constructed as $f^{s}=\left[f_{1}^{\left(s\right)},f_{2}^{\left(s\right)},\ldots ,f_{2V}^{\left(s\right)}\right]$. The log-transformation is used to normalize the elements of $f_{v}^{\left(s\right)}$

(3) $$f_{v}^{\left(s\right)}=\log\left(\mathrm{var}\left(w_{v}^{T}x\right)\right),\;v=1,2,\ldots ,2V,$$

where var(.) represents the variance. The CSP is used to extract a subset of features from each sub-band. The features obtained from each sub-band are concatenated as $F=[f^{1},f^{2},\ldots ,f^{s}]$ to create the feature vector for the particular trial, where $f^{s}$ represents the features of s sub-band and s=1,2,…,S (*S* represents all sub-bands together). The feature vector F is used to classify the MI tasks using SVM.

### 4.5. Classification

The proposed channel selection approach to MI Classification is evaluated using a support vector machine (SVM) classifier with a radial basis function (RBF) kernel. The effectiveness of the SVM is also compared with a linear discriminant analysis (LDA) classifier and an artificial neural network (ANN). The ANN is implemented with one hidden layer of size 10, sigmoid activation function, and one output layer of size 2 with linear function. The detailed description of SVM, LDA, and ANN is available here [46], [47], and [48], respectively.

## 5. Experimental Results

The performance of the proposed channel-selection-based method is assessed using the BCI competition III (IVA) dataset and the BCI competition IV(I) dataset. Right hand and right foot movement are two motor imagery tasks that are considered for classification. The signals of each channel of EEG data are decomposed into four sub-bands described in the Sub-band Decomposition Section. The CSP is applied to each sub-band of EEG signals to extract spatial information. From each of the four sub-bands, two sets of spatial filter pairs are extracted, producing four features. Thus 16 (4 feature × 4 sub-band)-dimensional feature vector is derived to feed to the classifier. The classification accuracy of the individual subject is evaluated using the k-fold (k = 5) cross-validation approach. The dataset is randomly divided into k equal groups. One group is designated for testing, while the rest is for training. The procedure is carried out over k times. The classification accuracy specified in Equation (4) is obtained by averaging the outcomes of the k iterations. With the use of the BCI Competition III (IVA) and IV (I) datasets, several experiments are conducted to illustrate the performance of the proposed approach method. Several performance metrics, Accuracy, Precision, Recall, and F1 score, are computed as follows:

(4) $$\mathrm{Accuracy}=\frac{\mathrm{TP}+\mathrm{TN}}{\mathrm{TP}+\mathrm{FP}+\mathrm{TN}+\mathrm{FN}}$$

(5) $$\mathrm{Precision}\;=\frac{\mathrm{TP}}{\mathrm{TP}+\mathrm{FP}}$$

(6) $$\mathrm{Recall}\;=\frac{\mathrm{TP}}{\mathrm{TP}+\mathrm{FN}}$$

(7) $$F1\;\mathrm{score}=2\times \frac{\mathrm{Precision}\times \mathrm{Recall}}{\mathrm{Precision}+\mathrm{Recall}}$$

where TP, TN, FP, and FN stand for true positive, true negative, false positive, and false negative, respectively. The classification performance of Dataset 1 and 2 with various classifiers is summarized in Table 3 and Table 4, respectively.

Table 3 illustrates that the SVM achieves the highest classification accuracy for individual subjects except ‘al’ and ‘ay’. The LDA performs better than that of SVM with these two subjects. The average classification accuracy of the SVM classifier outperforms ANN and LDA by 2.64% and 3.29%, respectively. Table 3 also demonstrates that for most of the cases, the F1 score, recall, and precision of the SVM classifier achieve higher performance. The mean value of the SVM classifier’s F1 score, recall, and precision is higher than the ANN and LDA classifiers. For subjects ‘aa’, ‘av’, and ‘aw’, SVM achieves the highest accuracy, F1 score, recall, and precision performance than ANN and LDA. The ANN achieves the highest recall for the subject ‘ay’, and the LDA obtains the highest accuracy, precision, and F1 score for the subject ‘ay’. Table 4 illustrates that SVM achieves the highest classification accuracy across all the subjects except ‘e’. It is noted that the best accuracy of subject ‘e’ is achieved by both ANN and LDA. The mean accuracy of SVM is 2.88% and 3.29% higher than that of ANN and LDA. The SVM classifier demonstrates superior performance compared with both the ANN and LDA classifiers in terms of mean accuracy, precision, recall, and F1 score across all the subjects. The implementation of the method is available at https://github.com/Shiam-Cse-Ru/EEG (accessed on 17 April 2024).

## 6. Discussion

**Figure 6:**
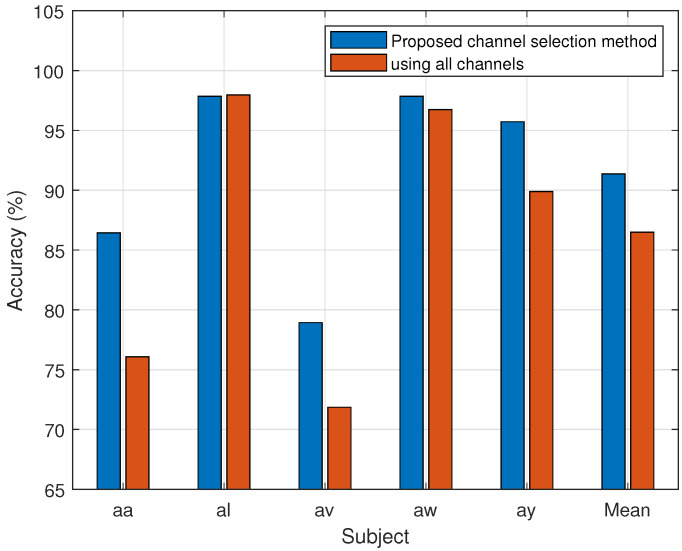
Comparison of the proposed channel selection method’s performance (accuracy) vs. all channels of Dataset 1.

**Figure 7:**
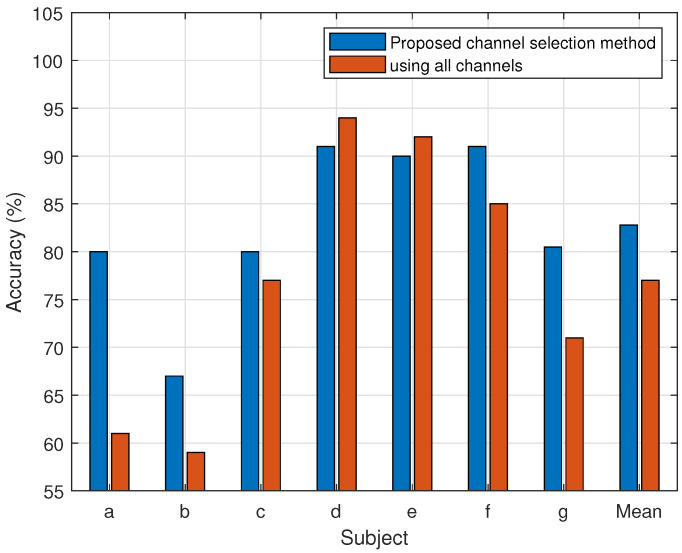
Comparison of the proposed channel selection method’s performance (accuracy) vs. all channels of Dataset 2.

Figure 6 and Figure 7 show the results of dataset 1 and 2, respectively, for the individual subjects in terms of classification accuracy for MI-BCI utilizing all channels, as well as the proposed channel selection method. All the channels are used to implement the conventional CSP-SVM technique. Performance with all channels is consistently worse than that of the proposed method. It has been noted that the channel selection strategy performs better than the average accuracy across all subjects and for each subject individually.

The proposed approach selects the least possible channels for each subject with maximum performance. The results of the experiment show that higher accuracy is achieved for a subject using fewer effective channels (see Figure 6 and Figure 7). The average classification accuracy of the proposed channel selection method with SVM is 4.86% greater than that of the use of all channels. In [49,50], the 30 channels are manually selected to classify the MI tasks. The information represented by EEG is very much subject-dependent. A uniform method of manual channel selection does not guarantee effectiveness in an intersubject scenario. The EEG data and subject-dependent channel selection approach are supposed to work better for MI task classification. Considering the underlying neurological assumptions, an entropy-based approach is implemented in this study to select an effective channel for BCI implementation. It is based on subject-specific EEG data.

**Figure 8:**
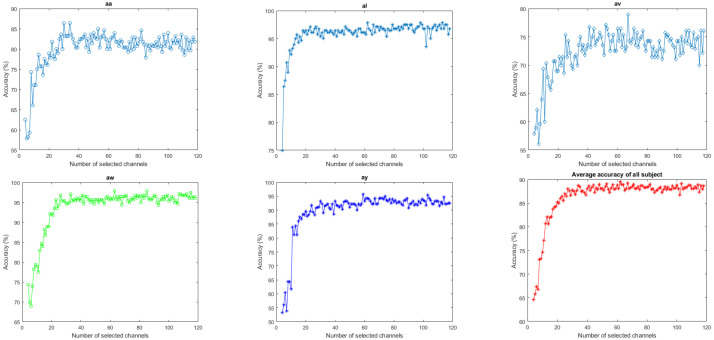
Individual subject performance (classification accuracy) and their average as a function of a number of selected channels (using an SVM classifier) of Dataset 1.

The number of channels used for the MI classification is one of the crucial variables affecting accuracy. Individual subject performance is shown in Figure 8 as a function of the number of effective channels selected by the proposed entropy-based approach. Although the classification accuracy of the majority of subjects initially improved with the number of channels, whereas the end performance is lower than the best accuracy reached with an optimal number of selected channels. It occurs because the use of fewer channels initially includes fewer features, leading to lower accuracy. The inclusion of a significantly higher number of channels of redundant information reduces the accuracy. With an effective number of selected channels, the highest accuracy is attained for each subject. In this experiment, the number of channels with the highest average subject-level accuracy is taken into account.

**Figure 9:**
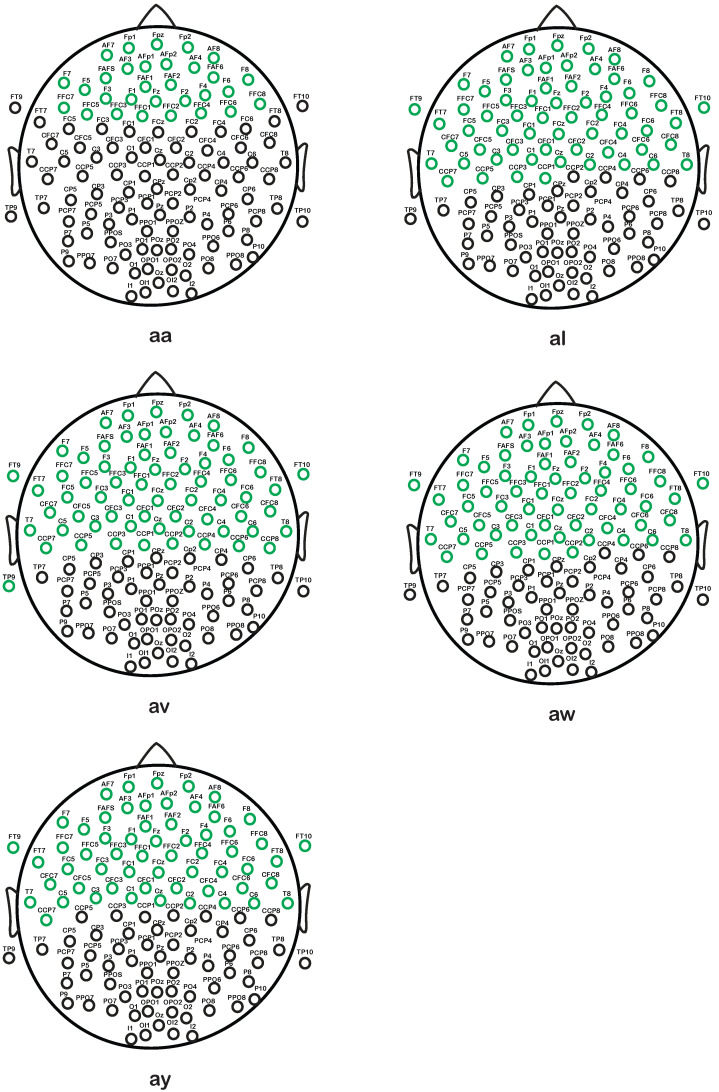
Distribution of the selected effective channels (green circles) of individual subjects of Dataset 1.

Figure 9 shows the selected effective channels by the proposed channel selection approach. It demonstrates how different combinations of channels are used to recognize the MI tasks. The distribution of the selected channels in the cerebral cortex’s motor regions is evident from Figure 9. The selected channels are 30, 62, 67, 63, and 59 for the subjects “aa”, “al”, “av”, “aw”, and “ay”, respectively, as shown in Figure 9. With the aforementioned carefully selected channels, the proposed method achieves the maximum classification accuracy.

The performance of the proposed method is compared with recent channel selection techniques, as illustrated in Table 5 and Table 6. The number of selected channels is indicated within the parentheses. It is noted that individual methods have selected different numbers of channels for different subjects. The challenging task is to fix up for the classification of motor imagery tasks with optimal performance. In comparison with previous techniques, the proposed method performs better in terms of accuracy across all the subjects. It is compared with the recently developed channel selection algorithm, such as dynamic channel relevance [30], channel selection using a correlation coefficient [25], CSP-R-MF [29], LRFCSP [31], CCS-RCSP [23], and FCCR [32] to understand its superiority. The dynamic channel relevance (DCR) score was utilized to find the effective EEG channels in [30] with a mean classification accuracy of 87.58%. The proposed method achieves the highest mean classification accuracy (91.36%) compared with other recently developed methods.

The correlation coefficient is used in [25] for selecting distinctive channels and achieved 88.62 % mean classification accuracy. Using the efficient channel selection strategy, the proposed method improves noticeable accuracy. It has been noted that the CCS-RCSP technique obtains a mean accuracy of 87.05% using correlation coefficients to pick MI-related channels. The technique described in [29], CSP-rank for multiple frequency bands (CSP-R-MF), selects the MI-relevant channels for each frequency band. They extracted features from those channels using the least absolute shrinkage and selection operator (LASSO) technique and obtained a mean accuracy of 85.87%. The proposed method outperforms the methods described in [25,30,51], CSP-R-MF [29], LRFCSP [31], CCS-RCSP [23], and FCCR [32], by 2.56%, 3.78%, 2.74%, 5.49%, 4.77%, 4.31%, and 5.46%, respectively, in terms of classification accuracy using Dataset 1. Table 6 shows the comparative performance of proposed method with recently developed methods using Dataset 2. It shows that the methods FCCR and CSP-R-MF consider only ‘a’, ‘b’, ‘f’, and ‘g’ subjects, while others consider all the subjects. The mean accuracy of the proposed method outperforms all the recently developed methods, as shown in Table 6. Considering the overall performance of MI task classification, the proposed information-theoretic EEG channel selection method is effective for BCI implementation. The traditional machine learning approaches SVM, LDA, and ANN are employed in this study rather than the deep learning methods. The proposed approach exhibits several advantages. The mentioned traditional methods are suitable for limited training data and less prone to overfitting. Less training data is more preferable for BCI implementation. The deep learning approach sometimes results in better performance, but it requires higher computational power and a high volume of training data. Such requirements make deep learning less preferable in the BCI paradigm. Additionally, this study focuses on effective EEG channel selection to enhance the MI classification performance. Thus, the proposed method provides a pragmatic and effective approach to implementing an effective BCI system. The experimental findings exhibit the effectiveness of the proposed approach to EEG classification. The results can lead to a broader acceptance and incorporation of BCIs into daily life applications like communication, rehabilitation, and other areas. The main limitation of the proposed channel-selection-based method is that it is subject-dependent and applicable to classify only two MI tasks. It is not tested with the data collected in a real real-world application environment.

## 7. Conclusions

In this study, an effective EEG channel selection approach is introduced to enhance motor imagery classification performance. The aim of the proposed method is to reduce redundant information by selecting effective channels. The use of a higher number of irrelevant EEG channels can increase the redundant information, which may cause the degraded performance of MI task classification. Effective channel selection is an efficient way to overcome the mentioned problems, leading to improved EEG classification accuracy. After the selection of potential channels, the EEG signals are decomposed into four sub0bands, and CSP-based features are extracted from individual sub-bands, yielding multiband features. The feature vector is derived by combining the features obtained from the sub-band signals. An SVM is employed to classify the MI tasks using the obtained feature vector. The proposed system improves the binary MI classification performance with two publicly available datasets compared with the state-of-the-art methods. The future extension of this work is to develop a method for subject-independent EEG channel selection and implementation for the multiclass MI classification paradigm.

## Figures and Tables

**Table 1:** Summary of the Dataset 1.

Dataset Name	BCI Competition III IVA
Subjects	aa, al, aw, av, ay
Number of Subjects	5
Channels	118
Sample Frequency	100 Hz
Classes	Left Hand, Right Hand, Foot
Tasks	Each subject completed any two of the classes
Visual Cues Duration	3.5 s
Total Trials	280 per subject (140 trials for each of the two classes)
Samples per Trial	350 (3.5 s × 100 Hz)

**Table 2:** Summary of the Dataset 2.

Dataset Name	BCI Competition IV Dataset I
Subjects	a, b, c, d, e, f, g
Number of Subjects	7
Channels	59
Sample Frequency	100 Hz
Classes	Left Hand, Right Hand, Foot
Tasks	Each subject completed any two of the classes
Visual Cues Duration	4 s
Total Trials	200 per subject (100 trials for each of the two classes)
Samples per Trial	400 (4 s × 100 Hz)

**Table 3:** Performance of the proposed method using SVM, ANN, and LDA classifiers in terms of classification accuracy (Acc.), precision (Prec.), recall (Rec.), and F1 score (F1) of dataset 1. Boldface denotes the best result.

Subjects	No. of Channels	Classifier											
		**SVM**				**ANN**				**LDA**			
		**Acc.**	**Prec.**	**Rec.**	**F1**	**Acc.**	**Prec.**	**Rec.**	**F1**	**Acc.**	**Prec.**	**Rec.**	**F1**
aa	30	**86.43**	**84.96**	**87.14**	**86.04**	83.75	84.38	82.16	83.26	81.43	80.73	82.29	81.50
al	62	97.86	97.24	**98.56**	97.90	98.14	**97.56**	97.67	97.61	**98.21**	97.29	99.29	**98.28**
av	67	**78.93**	**78.64**	**77.96**	**78.30**	71.21	73.12	65.04	68.84	68.57	68.12	72.57	70.27
aw	63	**97.86**	**96.67**	**97.88**	**97.27**	96.43	95.33	97.19	96.25	96.07	96.55	**97.86**	97.20
ay	59	95.72	93.98	95.36	94.67	94.07	94.16	**95.56**	94.85	**96.07**	**95.60**	95.00	**95.30**
**Mean**		**91.36**	**90.30**	**91.38**	**90.83**	91.08	88.91	87.52	88.16	88.07	87.66	89.40	88.51

**Table 4:** Performance of the proposed method using SVM, ANN, and LDA classifiers in terms of classification accuracy (Acc.), precision (Prec.), recall (Rec.), and F1 score (F1) of dataset 2. Boldface denotes the best result.

Subjects	No. of Channels	Classifier											
		**SVM**				**ANN**				**LDA**			
		**Acc.**	**Prec.**	**Rec.**	**F1**	**Acc.**	**Prec.**	**Rec.**	**F1**	**Acc.**	**Prec.**	**Rec.**	**F1**
a	23	**80.00**	78.30	78.78	78.54	79.00	73.08	**78.89**	75.87	78.50	**79.41**	76.43	77.89
b	31	**67.00**	**67.00**	**67.00**	**67.00**	61.80	62.44	61.00	61.71	59.50	58.88	62.00	60.40
c	41	**80.00**	**79.45**	**81.00**	**80.22**	73.90	74.44	74.20	74.32	72.00	71.43	74.00	72.69
d	46	**91.00**	**90.41**	91.00	**90.7**	88.20	87.79	89.10	88.44	89.50	87.38	**93.00**	90.10
e	39	90.00	90.68	90.00	90.34	**91.00**	**91.88**	90.00	90.93	**91.00**	90.04	**92.60**	**91.29**
f	31	**91.00**	**90.15**	**90.00**	**90.07**	85.95	88.69	83.00	85.75	86.50	84.75	89.00	86.82
g	17	**80.50**	79.25	**82.00**	**80.6**	79.50	79.39	80.40	79.89	79.50	**83.27**	77.00	80.01
**Mean**		**82.79**	**82.18**	**82.83**	**82.50**	79.91	79.67	79.51	79.56	79.50	79.31	80.58	79.89

**Table 5:** Performance comparison in terms of accuracy (%) with Dataset 1 using different cutting-edge channel-selection-based methods. Bold font is used to indicate the best outcome.

Methods	Subjects Accuracy (Number of Selected Channels)					Mean ± STD
	**aa**	**al**	**av**	**aw**	**ay**	
stdWC [51]	84.50 (16)	98.10 (19)	72.80 (12)	95.10 (13)	92.50 (20)	88.80 ± 9.10
DCRCC [30]	**93.60 (24)**	79.2 (33)	**94.6 (11)**	85.54 (26)	84.94 (31)	87.58 ± 6.46
CSCC [25]	89.29 (10)	**98.21 (7)**	73.47 (12)	92.86 (9)	89.29 (7)	88.62 ± 9.22
CSP-R-MF [29]	82.14 (12)	96.42 (12)	72.14 (12)	84.38 (12)	94.28 (12)	85.87 ± 9.83
LRFCSP [31]	83.93 (22)	96.42 (7)	74.49 (6)	88.84 (7)	89.29 (11)	86.59 ± 8.10
CCS-RCSP [23]	83.03 (42)	96.42 (33)	70.91 (52)	92.41 (14)	92.46 (67)	87.05 ± 10.28
FCCR [32]	78.57 (10)	**98.21 (10)**	72.45 (5)	87.05 (15)	93.25 (9)	85.90 ± 10.51
**Proposed Method**	86.36 (60)	97.51 (60)	76.20 (60)	**95.81 (60)**	**95.38 (60)**	**90.25 ± 8.98**

**Table 6:** Performance comparison in terms of accuracy (%) with Dataset 2 using different cutting-edge channel-selection-based methods. Bold font is used to indicate the best outcome.

Methods	Subjects Accuracy (Number of Selected Channels)							Mean ± STD
	**a**	**b**	**c**	**d**	**e**	**f**	**g**	
**Improved SFFS [52]**	69.00 (6)	63.00 (15)	87.00 (26)	94.00 (29)	**96.00 (19)**	65.00 (8)	72.00 (22)	78.00 ± 14.00
**FCCR [32]**	77.00 (14)	71.00 (9)				76.50 (9)	77.00 (18)	75.36 ± 2.53
**GSFS [53]**	75.00 (6)	**72.00 (13)**	**89.00 (11)**	78.00 (9)	84.00 (14)	78.00 (15)	83.00 (12)	79.80 ± 5.38
**CSP-R-MF [41]**	**81.50 (24)**	63.00 (24)				79.00 (24)	**87.50 (24)**	77.75 ± 9.06
**CSRI [54]**	72.80 (8)	66.18 (12)	83.72 (26)	**96.10 (13)**	83.50 (15)	76.33 (14)	87.33 (17)	80.85 ± 9.18
**Proposed Method**	80.00 (23)	67.00 (31)	80.00 (41)	91.00 (46)	90.00 (39)	**91.00 (31)**	80.00 (17)	**82.79 ± 8.73**

## Data Availability

The proposed model is evaluated with a benchmark dataset, namely the BCI Competition III and IV (IVA and I) datasets, which are publicly available. The dataset link is provided here www.bbci.de/competition/iii/ (accessed on 17 April 2024) and www.bbci.de/competition/iv/ (accessed on 17 April 2024).

## Abbreviations

The following abbreviations are used in this manuscript:

fMRI	Functional Magnetic Response Imaging
MEG	Magnetoencephalogram
EOG	Electrooculography
ERD	Event-Related Desynchronization
ERS	Event-related synchronization
CSP	Common Spatial Pattern
SVM	Support Vector Machine
ANN	Artificial Neural Network
LDA	Linear Discriminant Analysis
BSS	Blind Source Separation
RCSP	Regularized Common Spatial Pattern
RBF	Radial Basis Function
FBCSP	Filter-Bank Common Spatial Pattern
TSGSP	Time Constrained Sparse Group Spatial Pattern
SCSP	Sparse Common Spatial Pattern
DCR	Dynamic Channel Relevance
